# Supplementary material for: Unexplored diversity and potential functions of extra-chromosomal elements
Source: mSystems. 2025 Aug 19;10(9):e00175-25. doi: 10.1128/msystems.00175-25 (PMC12456017; doi:10.1128/msystems.00175-25)
Supplement: Supplemental figures — Fig. S1–S7. [file msystems.00175-25-s0001.pdf]

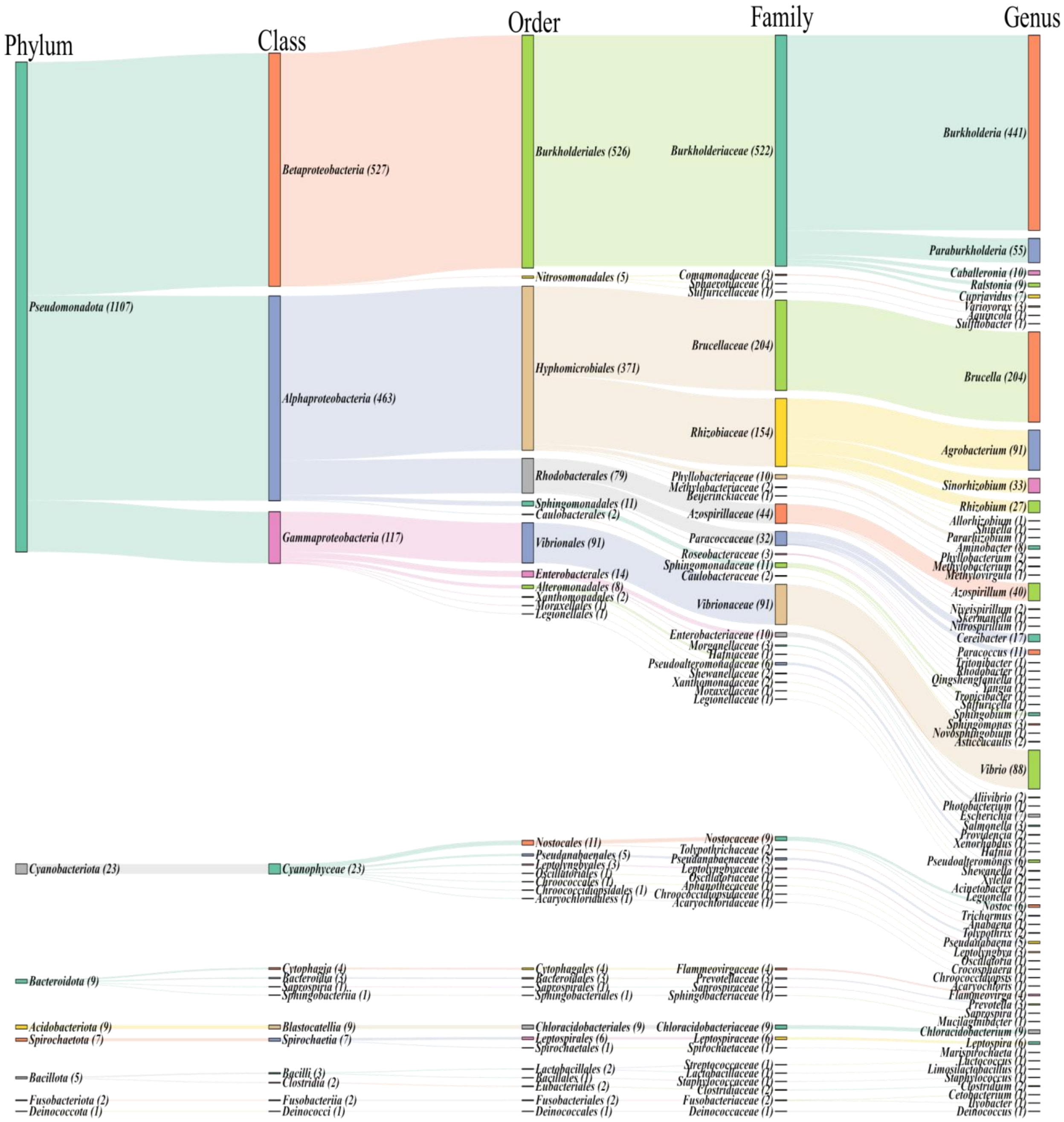

Extended Data Fig. S1 Phylogenetic distribution of chromid-carrying bacteria across different taxonomic levels. The numbers in parentheses indicate the quantity of genomes containing chromids within each specific taxonomic unit.

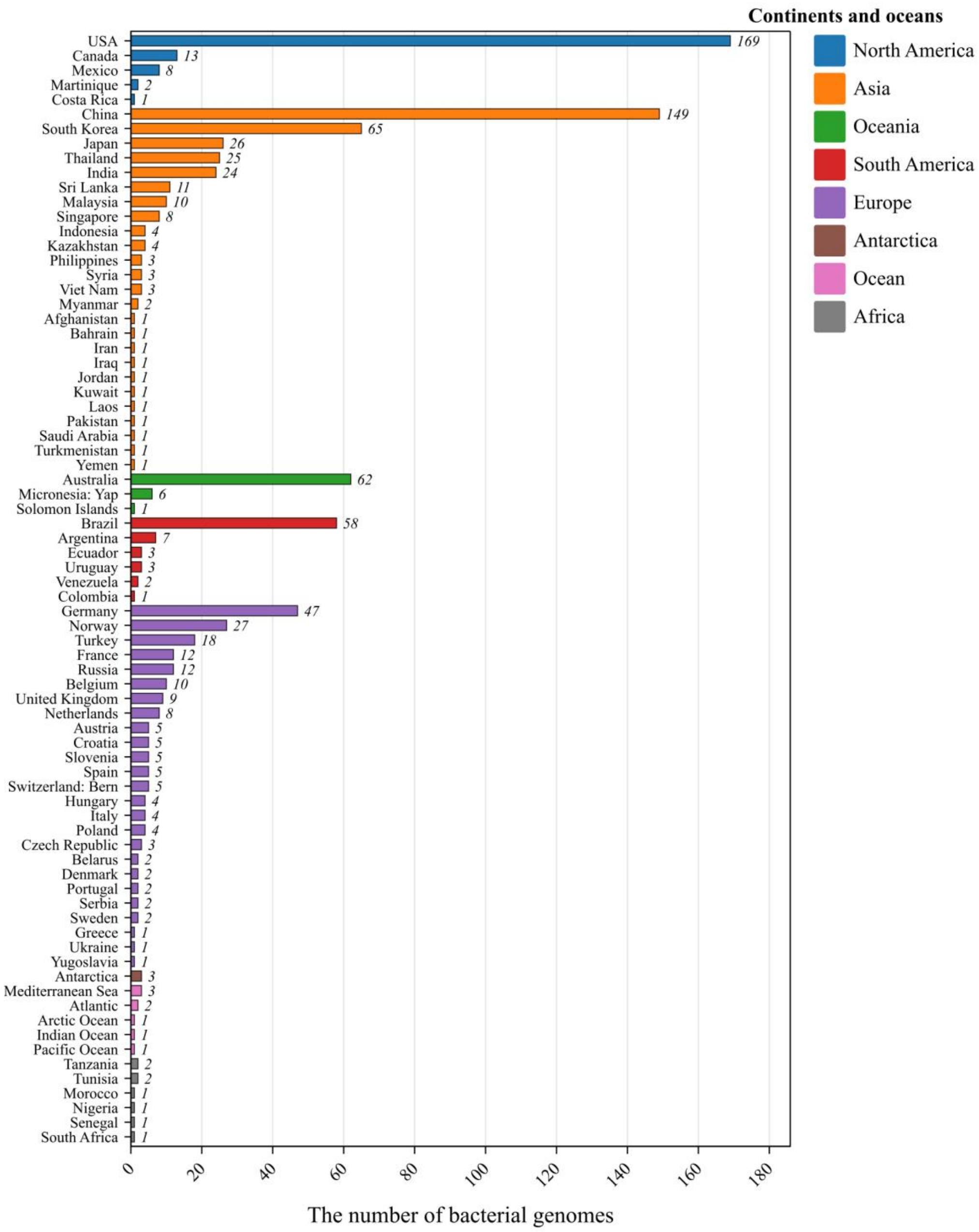

Extended Data Fig. S2 Distribution of chromids by country. showing only chromid-carrying bacterial genomes with available country information.

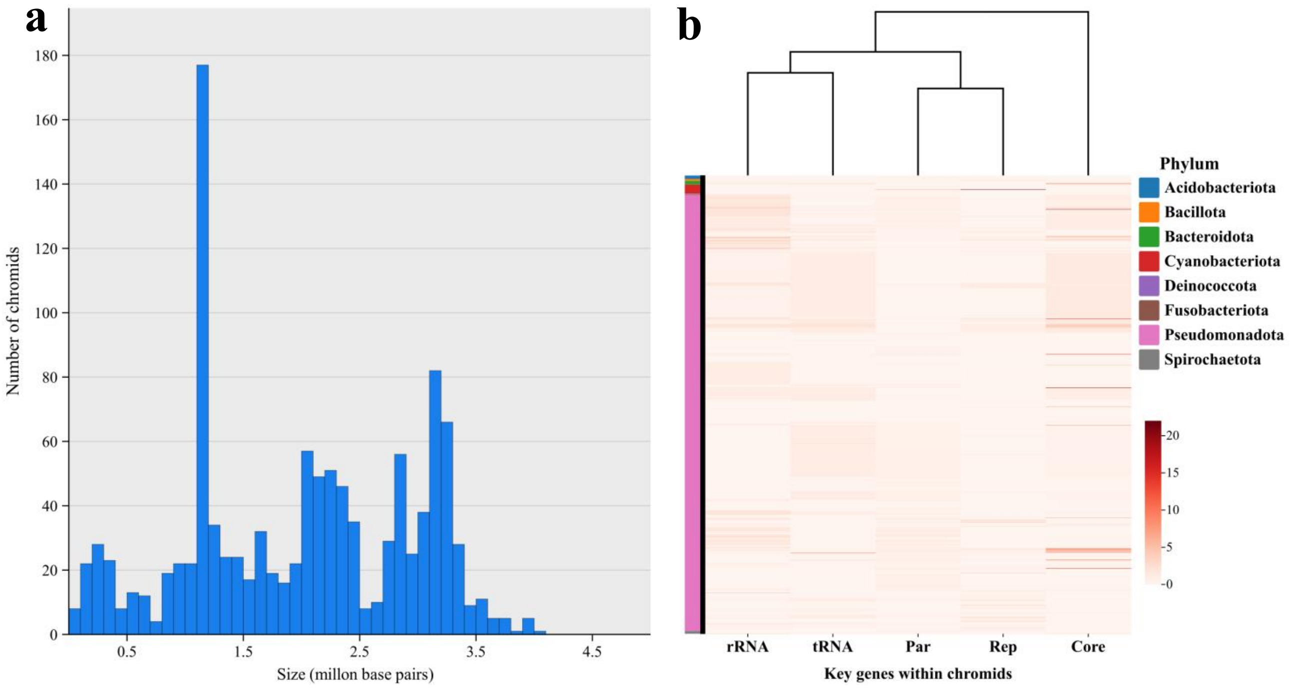

Extended Data Fig. S3 a, Size distribution of chromids. **b,** Heatmap showing the distribution of key genes within chromids.

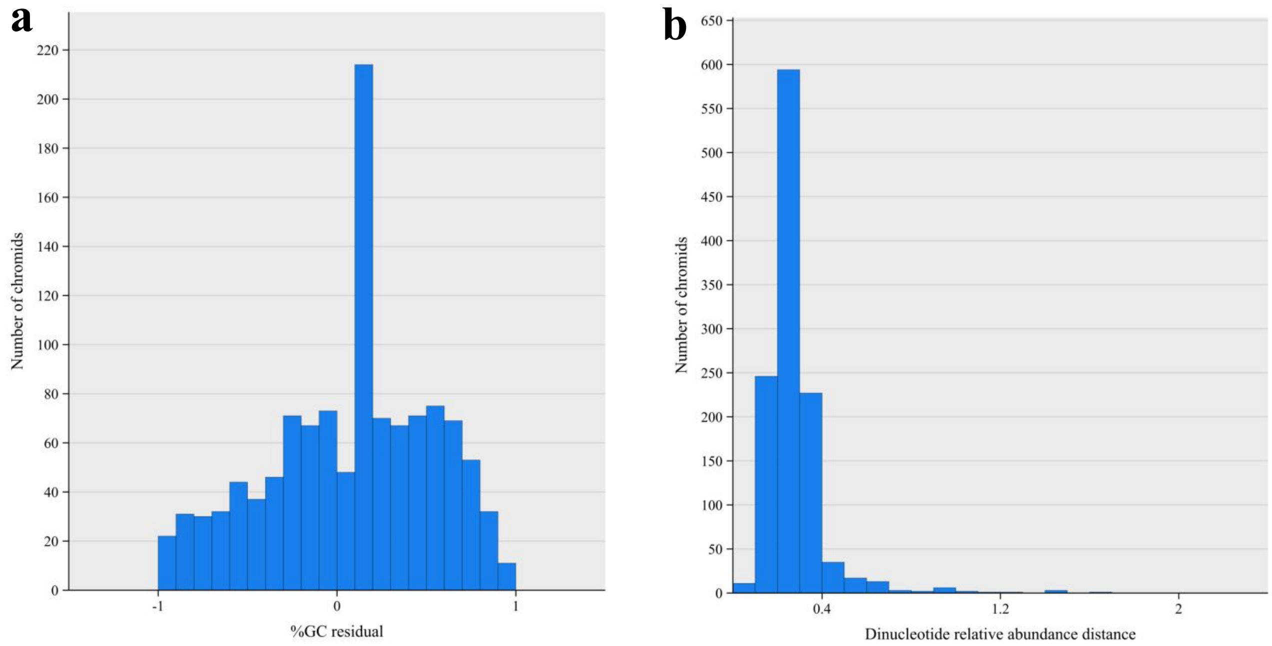

Extended Data Fig. S4 a, Difference in GC content between chromids and their corresponding chromosomes. **b,** Dinucleotide relative abundance distance between chromids and their corresponding chromosomes (right).

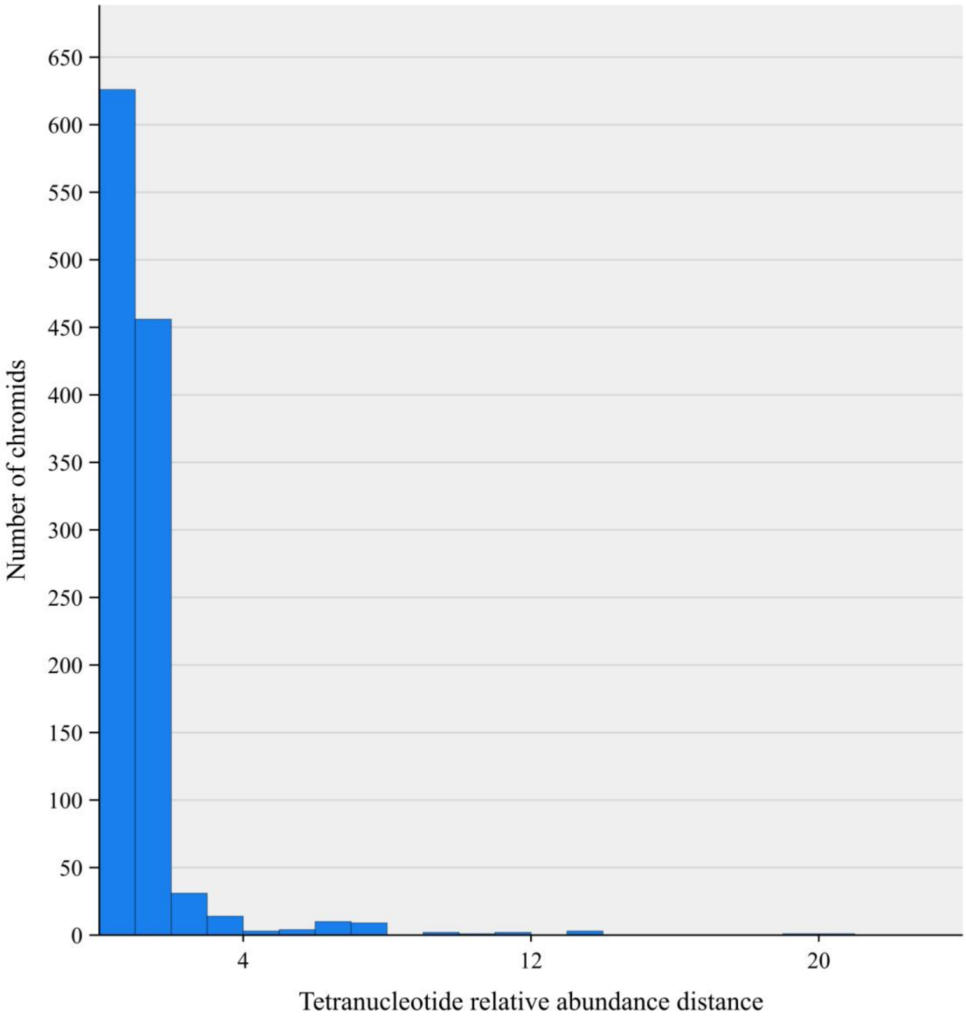

Extended Data Fig. S5 Tetranucleotide relative abundance distances between 1,163 chromids and their corresponding bacterial main chromosomes from previous studies.

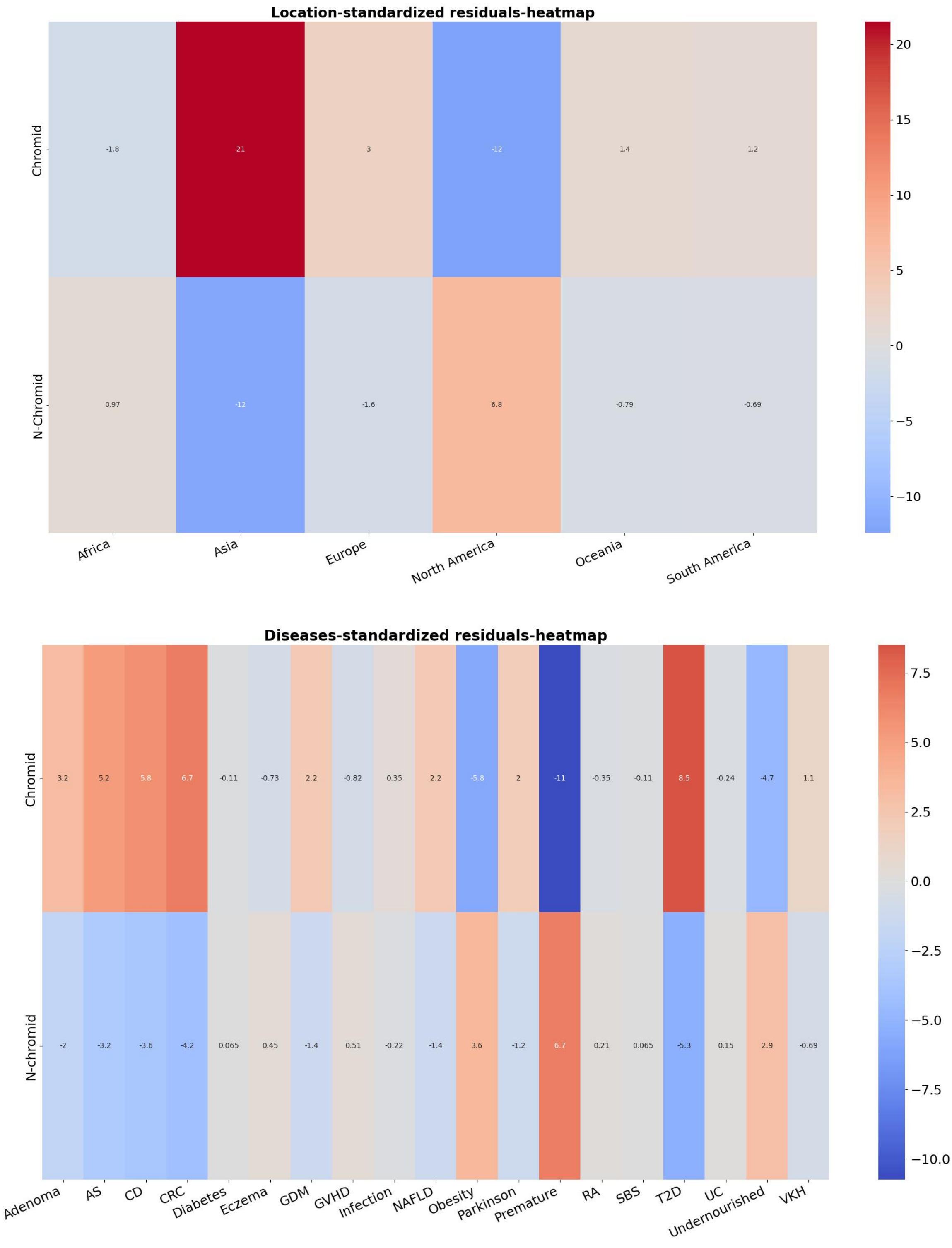

Extended Data Fig. S6 Heatmap of standardized residuals illustrating the impact of host geographical location and disease type on the distribution of chromid-carrying bacteria. AS = Ankylosing Spondylitis; CD = Crohn's Disease; CRC = Colorectal Cancer; GDM = Gestational Diabetes Mellitus; GVHD = Graft-versus-host Disease; NAFLD = Non-alcoholic Fatty Liver Disease; RA = Rheumatoid Arthritis; SBS = Short Bowel Syndrome; T2D = Type 2 Diabetes; UC = Ulcerative colitis; VKH = Vogt-Koyanagi-Harada disease. Obesity was defined as Body Mass Index (BMI) > 30 kg m^{-2}.

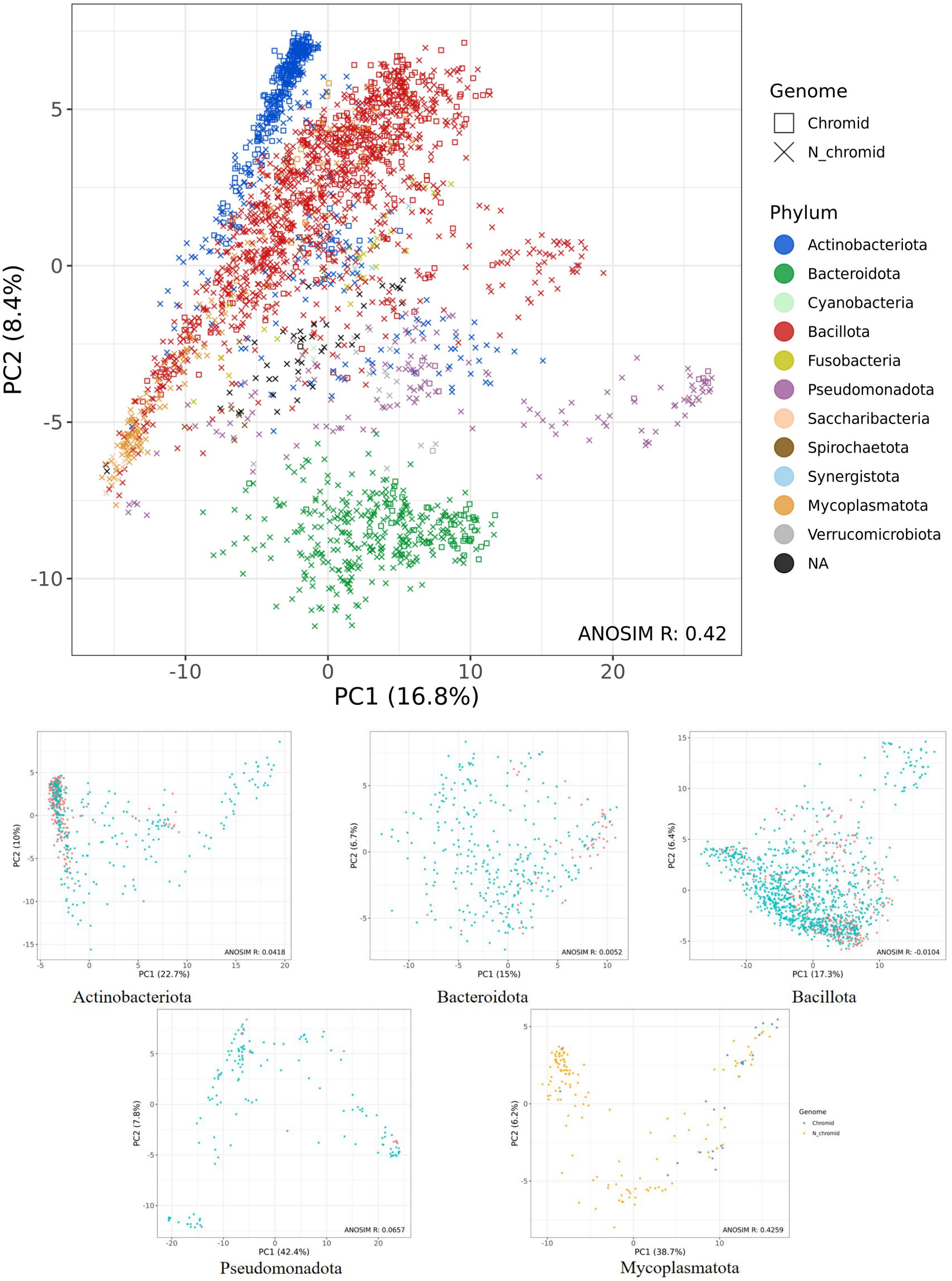

Extended Data Fig. S7 The Chromid-carrying bacteria secies have a distinct functional capacity. a, Principal component analysis (PCA) based on GPs of the chromid-carrying bacteria (n = 573 genomes) and the non-chromid-carrying bacteria (n = 1,932 genomes) coloured by phylum.**b,**PCA based on GPs of the 573 h_chromid_genomes and the 1,932 no_chromid_ genomes for the five most prevalent phyla.
